# Molecular and morphological alterations in uninjured skin of streptozotocin‐induced diabetic mice

**DOI:** 10.1590/1414-431X2023e12212

**Published:** 2023-01-27

**Authors:** T.P. Prado, J. Morari, E.P. Araújo

**Affiliations:** 1 Faculdade de Enfermagem, Universidade de Campinas, Campinas, SP, Brasil; 2 Laboratório de Sinalização Celular, Universidade de Campinas, Campinas, SP, Brasil; 3 Centro de Pesquisa em Obesidade e Comorbidades, Universidade de Campinas, Campinas, SP, Brasil

**Keywords:** Diabetes, Skin, Homeostasis, Inflammation, Growth factors, Streptozotocin

## Abstract

Diabetes affects every tissue in the body, including the skin. The main skin problem is the increased risk of infections, which can lead to foot ulcers. Most studies evaluating the effects of diabetes on the skin are carried out in wound healing areas. There are fewer studies on uninjured skin, and some particularities of this tissue are yet to be elucidated. In general, cellular and molecular outcomes of diabetes are increased oxidative stress and lipid peroxidation. For our study, we used C57BL/6 mice that were divided into diabetic and non-diabetic groups. The diabetic group received low doses of streptozotocin on 5 consecutive days. To evaluate the effects of hyperglycemia on uninjured skin, we performed morphological analysis using hematoxylin/eosin staining, cellular analysis using Picrosirius red and Nissl staining, and immunostaining, and evaluated protein expression by polymerase chain reaction. We confirmed that mice were hyperglycemic, presenting all features related to this metabolic condition. Hyperglycemia caused a decrease in interleukin 6 (*Il-6*) and an increase in tumor necrosis factor alpha (*Tnf-α*), *Il-10*, *F4/80*, tumor growth factor beta (*Tgf-β*), and insulin-like growth factor 1 (*Igf-1*). In addition, hyperglycemia led to a lower cellular density in the epidermis and dermis, a delay in the maturation of collagen fibers, and a decrease in the number of neurons. Furthermore, we showed a decrease in *Bdnf* expression and no changes in *Ntrk2* expression in the skin of diabetic animals. In conclusion, chronic hyperglycemia in mice induced by streptozotocin caused disruption of homeostasis even before loss of skin continuity.

## Introduction

Currently, diabetes mellitus (DM) affects more than 460 million people worldwide, a number that is expected to grow to 578 million by 2030 (1). DM impacts every structure of the body, including the skin (2). More than two-thirds of diabetic people are expected to have a skin problem at some point (3). The greatest risk factors are poor glycemic control and the duration of diabetes (4). The common causes of skin complications are related to an increase in both oxidative stress and lipid peroxidation (5). Lipid peroxidation is a harmful process mediated by free radicals, which cause polyunsaturated fatty acids in cell membranes to degrade to lipid hydroperoxides, which in turn cause cellular damage (6). There is also an increase in pro-inflammatory gene expression and transcription factors (5). Moreover, reductions in the hydration of the stratum corneum, lamellar body production, and expression of antimicrobial peptides have also been associated with DM (7).

The main clinical manifestations are premature aging of the skin (2) and the increased risk of secondary infections, such as those that occur in diabetic foot ulcers (4). Diabetic foot ulcers are among the most common complications of patients with DM due to impaired wound healing (4,8). They are one of the most common causes of osteomyelitis of the foot and lower extremity amputations (4,8). In fact, cutaneous complications are known to occur during the course of the disease, but they can also be the earliest manifestation of diabetes or even precede the diagnosis (8). However, there are aspects that need to be elucidated regarding the deleterious effects of chronic hyperglycemia on the skin of diabetic subjects.

Most studies are conducted in the area of wound healing. However, here, we studied the effects of diabetes on uninjured skin, with the purpose of revealing possible alterations preceding skin disruption that may interfere with the healing process of cutaneous wounds. Therefore, we used streptozotocin (STZ)-induced diabetic mice. STZ is an antibiotic that promotes complete or partial destruction of pancreatic β cells and is commonly used experimentally to produce a type 1 diabetic animal model (9). We divided C57BL/6 mice into two groups, diabetic and non-diabetic, and we performed hematoxylin/eosin, picrosirius red, and Nissl staining. In addition, we conducted immunostaining and polymerase chain reaction analysis to evaluate transcripts of cytokines, growth factors, receptors, and enzymes (*IL-10, IL-1β, IL-6, F4/80, ITGAα-5, TGF-1β, FGF-1, IGF-1, VEGF-α, α-SMA, MMP-9, BDNF, TRK2*) that are known to be involved in the wound healing process and skin homeostasis (10).

A better understanding of the etiological factors that lead to the development of cutaneous complications associated with diabetes could help advance new therapies and could help health professionals make a more accurate diagnosis and provide better treatment.

## Material and Methods

### Experimental animals

Eight-week-old male isogenic C57BL/6J mice from the University of Campinas Breeding Center (Brazil) were separated and randomized into two groups. The diabetic group received five low intraperitoneal (*ip*) doses of SZT (60 mg/kg) on consecutive days and the non-diabetic mice received citrate buffer. Initially, we evaluated eight mice from the non-diabetic group and 12 mice from the diabetic group. They were used for the physiological experiments and then for the collection of tissues, ending up with six mice per group, from a total of 40 mice. Diabetic mice die easily, resulting in the need to repeat several experiments. Four weeks after diabetes induction, the glycemic level was measured, and mice showing a glycemic level ≥250 mg/dL were included in the study. Mice were kept in a pathogen-free environment in separate enclosures on a 12-h light/dark cycle; the room temperature was kept at 21°C. Rodent chow and water were offered *ad libitum*. For surgical intervention, mice were sedated by *ip* inoculation with ketamine hydrochloride (80 mg/kg body weight) and xylazine chlorhydrate (8 mg/kg body weight). We applied a humanized method of euthanasia by deepening the anesthesia and cervical dislocation. All experiments were performed in accordance with NIH standards. The methods used were certified by the Animal Ethical Committee at the University of Campinas, Brazil (Certificate of Approval No. 5635-1/2020).

### Glucose evaluation, ipGTT, and ipITT

After 4 h of fasting, an intraperitoneal glucose tolerance test (ipGTT) and insulin tolerance test (ipITT) were performed on non-anesthetized mice, using blood collected from the caudal vein. The blood glucose levels were measured with an Optium Xceed Diabetes Monitoring System OptiumTM mini handheld glucometer using suitable test strips (Abbott Laboratórios, Brazil). For the ipGTT, a solution of 20% glucose (2.0 g/kg body weight) was administered into the peritoneal cavity. Blood samples were collected from the tail vein at 30, 60, 90, and 120 min post-glucose administration for the determination of glucose concentrations. The area under the curve (AUC) was defined using these values. To perform the ipITT, after collecting a fasting sample (time 0), the mice were injected *ip* with insulin (1.5 IU/kg body weight). Blood glucose measurements were taken at 5, 10, 15, 20, 25, and 30 min. The constant rate of glucose disappearance (kITT) was established using the following formula: 0.693/half-life. The glucose half-life was calculated from the slope of the least-squares evaluation of the blood glucose concentrations during the linear period of decay (time 5-15 min) (11). Mice that had hypoglycemia lower than 20 mg/dL had an *ip* glucose injection and were excluded from testing.

### Food and water intake and body weight evaluation

Body mass and food and water intake were recorded over 4 weeks. Mice were deprived of food and water for 4 h and then reintroduced to standard chow and water. Food intake was determined by calculating the difference between the weight of the provided food and the weight of food remaining at the end of 7 days. Water consumption was also determined the in same manner (in mL). Body weight was evaluated in non-anesthetized mice once a week. Food intake, water intake, and body weight were assessed daily until the end of the experimental period. All measurements were performed on the same day of the week and same period of the day. In the fourth experimental week, mice were subjected to the ipGTT and 2 days later, to the ipITT.

### Tissue collection for PCR

A skin biopsy was performed with a 6-mm metallic punch in the middle of the dorsal region. Samples were homogenized/blended in 1000 µL of phenol buffer and TRIzol reagent (Invitrogen, Brazil) using a Polytron PT-DA 12 / 2EC-E157 disperser (Kinematica, Switzerland) at maximum speed (1000 rpm) for 30 s. RNA extraction was performed with 200 µL of chloroform (Invitrogen) and precipitated using 500 µL of isopropanol (Invitrogen). The pellet was washed with increasing concentrations of ethanol (70 and 100%) and resuspended in deionized water at a concentration of 3000 ng/μL. RNA was quantified using a microplate spectrophotometer (ENON, Biotek, USA). Complementary DNA (cDNA) was produced from 2 μg total RNA using the High-Capacity cDNA Reverse Transcription Kit (Applied Biosystems, USA) in the Thermo cycler QxDX (Bio-rad, France).

### Transcript expression by real-time PCR

PCR reactions were achieved in real time using the TaqMan^TM^ system (Applied Biosystems). The Rpl0 gene (TaqMan^TM^ - Applied Biosystems) was used as the endogenous control for the reaction to which the transcripts of the target gene in different samples were standardized. Next, in the assessment of the amplification efficiency, a scatter plot was created to describe the series of concentrations for which the system was efficient. Primers for the target genes were obtained from Applied Biosystems against tumor necrosis factor (TNF)-α (Mm00443258_m1), interleukin (IL)-10 (Mm01288386_m1), IL-1β (Mm00434228_m1), IL-6 (Mm00446190_m1), F480 (Mm00802529), ITGα-5 (Mm164148797), TGF-1β (Mm.PT.58.43.479940), FGF-1 (Mm.PT.56a.41158563), IGF-1 (Mm.PT.58.5811533), VEGF-α (Mm.PT.58.14200306), α-SMA (Mm157808773), MMP-9 (Mm. 00442991_m1), BDNF (Mm.PT.58.13575048), and NTRK2 (Mm.PT.58.42284287). For the quantification of transcripts, the reactions were carried out in duplicate, each containing 3.0 µL of LuminoCt^®^ qPCR ReadyMix™ (Sigma-Aldrich, USA), 0.25 µL of primers and probe solution, 0.25 µL of water, and 4.0 μL of cDNA (40 ng). The negative control used 4.0 μL of water in place of cDNA. The cycling conditions were as follows: 95°C for 2 min, then 40 cycles of 95°C for 5 s, and 60°C for 30 s. The relative gene expression values were obtained by assessing the outcomes in StepOnePlus™ Software v2.3 (Applied Biosystems). Gene expression was recorded as fold-change and calculated considering the variation in gene expression compared to the control group (non-diabetic), normalized to the endogenous control (Rpl0). Fold-change = 2^- (gene QT mean - endogenous QT mean) / 2^- (QT mean - QT mean of non-diabetic group).

### Microscopy

Samples were fixed by immersion in formaldehyde and treated with different concentrations of alcohol (70, 80, 95, and 100%), followed by xylol and paraffin, and ultimately fixed in paraffin blocks that were subsequently segmented at 5-µm intervals and placed on microscope slides pre-treated with poly-L-lysine. Then, samples were stained with hematoxylin and eosin. The sections were incubated with hematoxylin for 30 s, bathed in distilled water, incubated for 30 s with eosin, cleaned in purified water, and dehydrated. The slides were placed on Entellan^®^ (Merck Millipore, USA), and the images were digitally taken under bright field microscopy (Zeizz Axiophot, fluorescent-microscope, USA). Picrosirius red staining was applied to examine collagen fibers. For this, sections were immersed in 100% xylene, followed by hydration in 100, 95, and 70% ethanol before being incubated in 0.1% picrosirius red (EasyPath, Brazil) for 1 h, rinsed with water, and incubated in Carazzi's hematoxylin for 4 min, successively, at room temperature. The double refraction patterns were evaluated by microscopy (Nikon^®^ Corporation, Japan), using C-PL polarized filters. The stained collagen fibers were analyzed using Image-Pro Plus (v4; Media Cybernetics Inc., USA), and were either bright green (type III collagen-like) or red (type I collagen-like). Using the Image J (http://imagej.nih.gov/ij/) and Orientation J plug-in (Biomedical Image Group, EPFL, Switzerland), we measured the quantities, organization, and density of collagen. For these analyses, five representative areas (800×800 pixels) were randomly chosen in each 8-bit image (1920×1080 pixels) (12). For the Nissl assay, after fixation with formaldehyde, the samples were dehydrated in progressive concentrations of sucrose in PBS solution (20 and 30%), then blocked in Tissue-Tek polymer (Sakura Finetek, USA), and sectioned at 10.0 µm intervals in a cryostat. The slides were immersed in a solution of 2% toluidine blue for 1 min and fixed with Entellan^®^. For immunofluorescence analysis, deep-frozen skin tissue samples were cut into transverse sections (10 μm). After blocking with Donkey Serum (D9663; Merck KGaA, Germany) in BSA 0.3% solution, specimens were labeled with anti-BDNF primary antibodies (sc-65513; Santa Cruz Biotechnology, USA). Thereafter, the sections were incubated with Alexa 546 conjugated IgG secondary antibody and TO-PRO™-3 Iodide (642/661) (Thermo Fisher Scientific, USA) for nuclear staining. The images were captured with an upright Zeiss LSM780-NLO Confocal Microscope (Carl Zeiss AG, Germany).

### Statistical analysis

Data are reported as means±SD or SE. For the statistical analysis, the Levine test was first used to check for the homogeneity of variance. To compare the means between unmatched groups, if the values followed a Gaussian distribution, we used the unpaired Student's *t*-test for independent samples. In all cases, the level of significance considered sufficient to reject the null hypothesis was 5% (P≤0.05). Data was evaluated using the Graph Prism 8.0.1 software (GraphPad, USA).

## Results

### Streptozotocin-induced diabetic mice revealed consistent physiological changes

**Figure 1:**
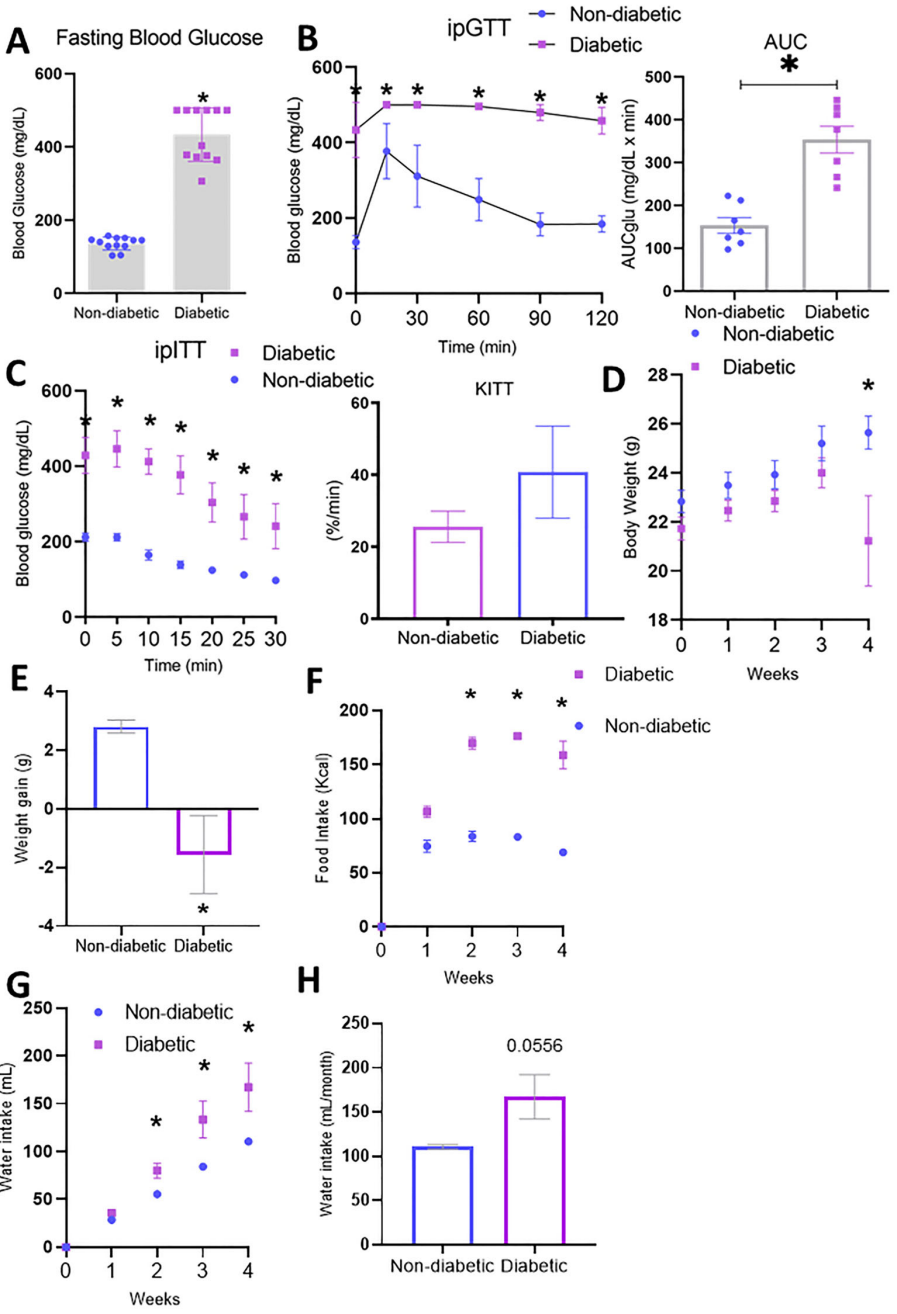
Metabolic parameters in diabetic and non-diabetic mice. **A**, Fasting blood glucose. **B**, Intraperitoneal glucose tolerance test (ipGTT) and area under the curve (AUC) of blood glucose. **C**, Intraperitoneal insulin tolerance test (ipITT) and the rate constant (KITT) of glucose decay, considering the slope of plasma glucose concentration 3-15 min after administration of intravenous insulin. **D**, Weight gain over 4 weeks. **E**, Cumulative body weight gain. **F**, Food intake over 4 weeks. **G**, Evaluation of weekly water intake. **H**, Cumulative water consumption over 4 weeks. Error bars represent standard error of the mean (SE) across at least six independent samples. *P≤0.05 diabetic *vs* non-diabetic groups (Student's *t*-test).

The diabetic mice showed significantly elevated blood glucose compared with non-diabetic mice (Figure 1A and B). However, based on the ipITT, the mice did not develop insulin resistance, even though they had high blood glucose levels from the beginning to the end of the test (Figure 1C). As expected, although the diabetic mice had a higher food intake compared with the non-diabetic animals, they lost weight, which was probably due to the higher catabolism caused by hyperglycemia (Figure 1D-F). In addition, as we also predicted, they had a higher water intake compared to non-diabetic animals (Figure 1G and H).

### Skin of ZTC-induced diabetic mice showed modulated expression of cytokines, growth factors, and macrophage markers

**Figure 2:**
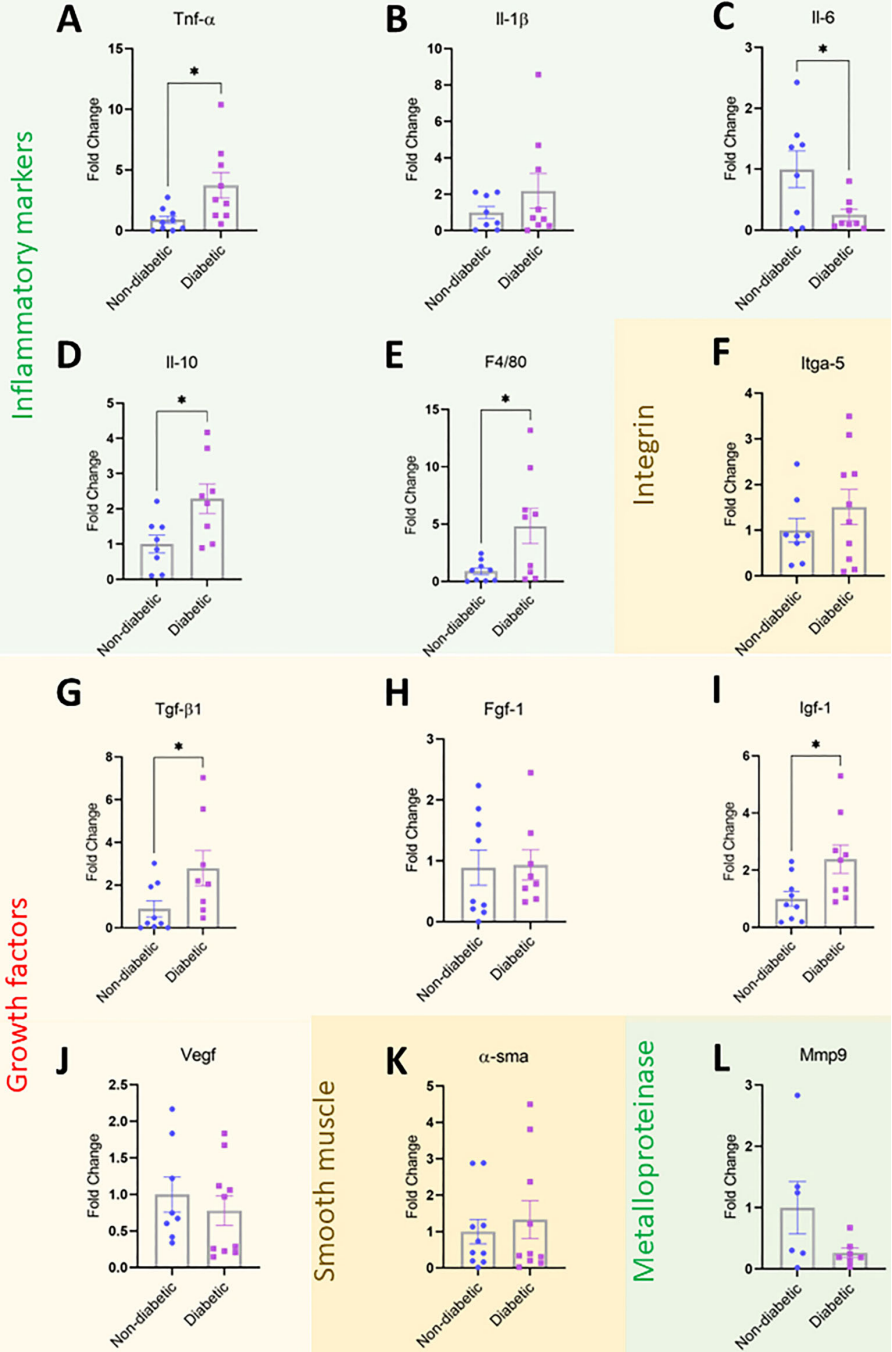
Determination of mRNA transcripts in diabetic and non-diabetic mice. **A**, *Tnf-α*; **B**, *Il-1β*; **C**, *Il-6*; **D**, *Il-10*; **E**, *F4/80*; **F**, *Itga-5*; **G**, *Tgf-β1*; **H**, *Fgf-1*; **I,** *Igf-1;* **J**, *Vegf*; **K**, *α*-*Sma*; and **L**, *Mmp-9*. Data are reported as fold-changes between the diabetic and non-diabetic groups. Error bars represent standard error of the mean (SE) across at least six independent samples. *P≤0.05 (Student's *t*-test).

We showed a significant increase in gene expression of *Tnf-α, Il-10*, and *F4/80* (Figure 2A, D, E), and a decrease in *Il-6* (Figure 2C). Moreover, we evaluated *Itga5*, which belongs to a family of proteins that play important roles in cytoskeletal organization and cell migration, proliferation, and survival. However, there was no significant change in this integrin (Figure 2F). In addition, we evaluated growth factors and other proteins important for skin homeostasis and tissue repair. There were significant increases in the expression of *Tgf-β1* and *Igf-1* (Figure 2G and I) in hyperglycemic mice. No significant changes were observed for *Il-1β*, *Fgf-1*, *Vegf*, *α-Sma*, or *Mmp-9* (Figure 2B, H, J-L).

### STZ-induced diabetic mice had a lower cellular density in the epidermis and dermis

**Figure 3:**
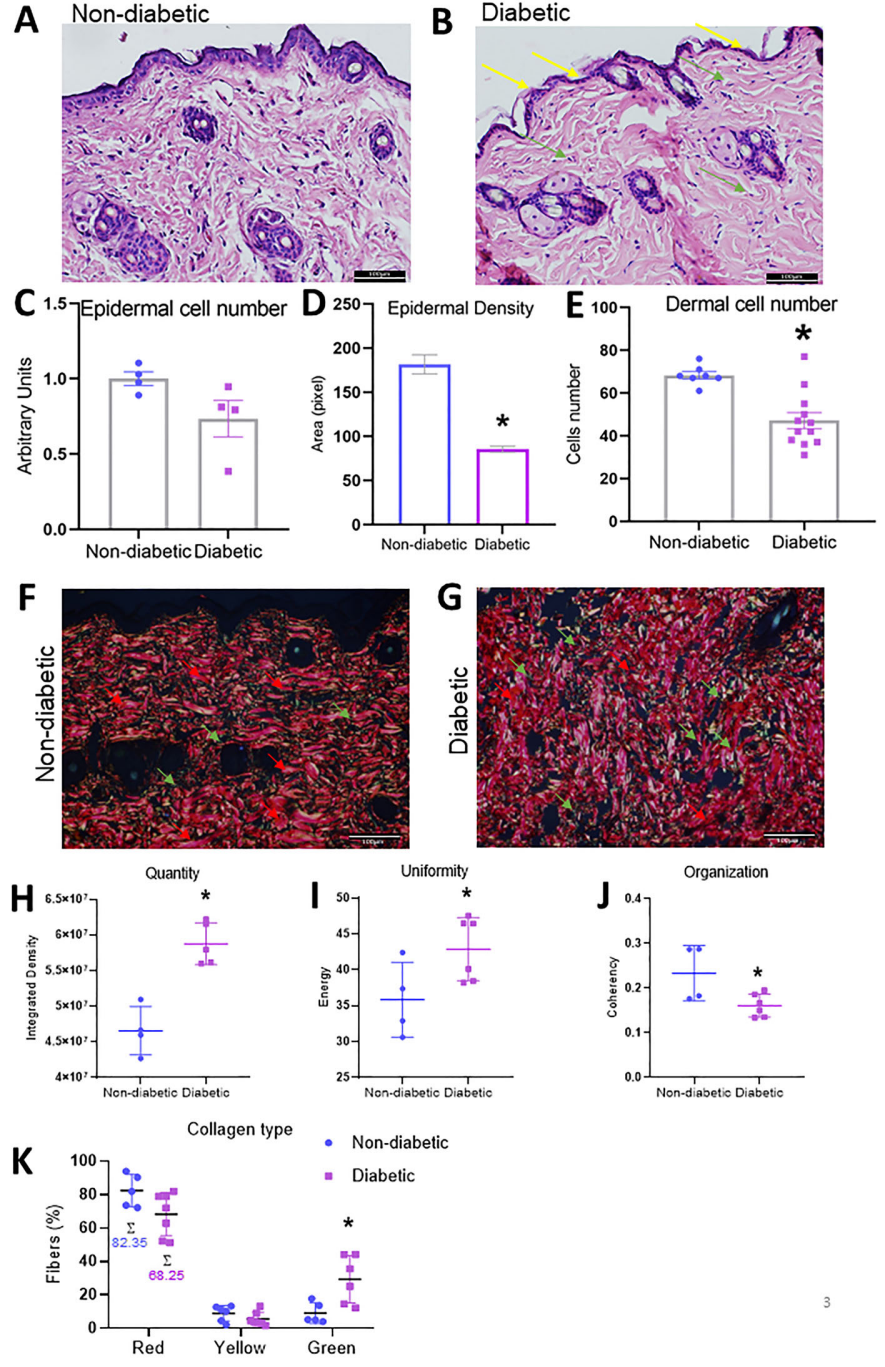
Morphological and collagen evaluation of skin. **A** and **B**, Morphological features revealed by hematoxylin and eosin staining of skin from non-diabetic and diabetic mice, respectively (20×, scale bar 100 μm). The yellow arrows show the thinner epidermal thickness in diabetic animals. **C**, Fold-change in epidermal cell number. **D**, Fold-change of epidermal density. **E**, Fold-change of dermal cell number. **F** and **G**, Picrosirius red staining showing collagen deposition in non-diabetic and diabetic mice (scale bar 100 μm). **H**, **I**, and **J**, Collagen fiber analyses (quantity, uniformity, and organization, respectively). **K**, Type I and type III collagen fiber analyses; absorbance of red, yellow, and green values. Error bars represent standard error of the mean (SE) across at least four independent samples. *P≤0.05 diabetic *vs* non-diabetic groups (Student's *t*-test).

We analyzed skin morphology of diabetic and non-diabetic mice using H/E staining (Figure 3). The epidermis of diabetic mice had a lower cellular density (Figure 3B and D). Furthermore, keratinocytes were thinner and more elongated in diabetic mice than in non-diabetic mice (yellow arrows). There was also a significant decrease in cellularity in the dermis of diabetic mice (green arrows) (Figure 3B and E).

### Skin of STZ-induced diabetic mice contained immature collagen

Collagen in the skin of non-diabetic mice was dense, with wider fibers, and better organized with intertwined fibers compared with diabetic mice (Figure 3F and G). There was more collagen in diabetic mice; however, fibers showed greater uniformity and a more random organization than in non-diabetic mice (Figure 3H-J). Moreover, the diabetic mice had more green collagen fibers according to the birefringence in polarized light, indicating mostly immature collagen, compared to non-diabetic mice (Figure 3K).

### STZ-induced diabetic mice had lower *BDNF* expression and fewer neurons

**Figure 4:**
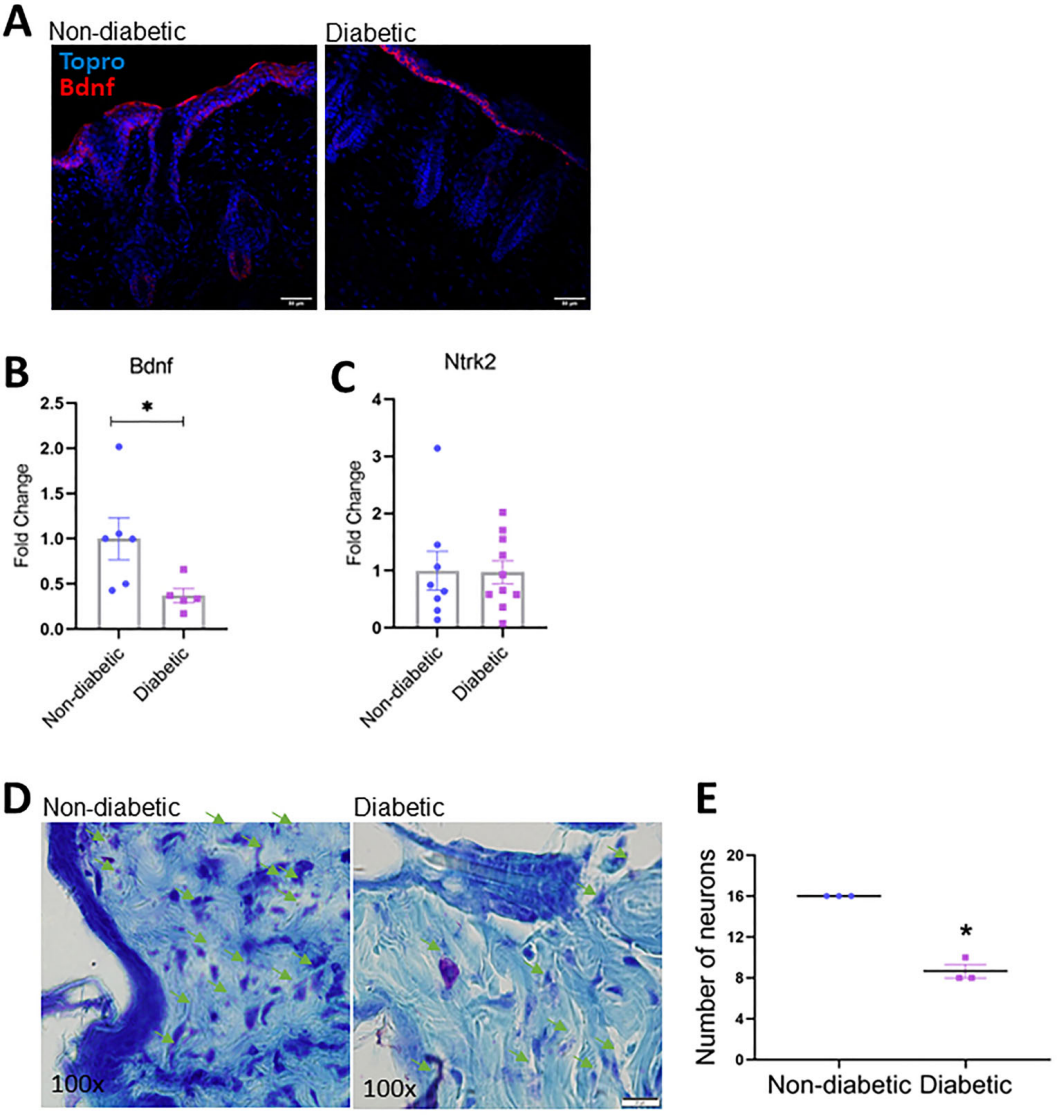
Evaluation of Bdnf, Ntrk2, and peripheral neurons in the skin of diabetic mice. **A**, Immunohistochemistry showing BDNF in red and cellular nucleus in blue (Topro) (scale bar 100 μm). **B**, Evaluation of Bdnf and **C**, Ntrk2 transcripts. **D**, Nissl staining showing purple staining of the nucleus of neurons (scale bar 100 μm). **E**, Number of neurons. Error bars represent standard error of the mean (SE) across at least six independent samples. *P≤0.05 diabetic *vs* non-diabetic groups (Student's *t*-test).

We found a decrease in BDNF protein and gene expression and no changes in its receptor Ntrk2 in the skin of diabetic mice (Figure 4A, B, C). In addition, we observed a significant reduction in the number of peripheral neurons by Nissl staining (Figure 4D and E).

## Discussion

In the present study, we found that STZ-induced diabetes resulted in a significant increase in blood glucose, as mice developed severe hyperglycemia with concentrations reaching 400 mg/dL. Repeated administration of five low doses of STZ resulted in cellular and humoral immune reactions against β cells following subsequent infiltration of macrophages and lymphocytes, leading to β-cell lysis and type 1 diabetes (9). Despite the hyperglycemia, mice with STZ-induced diabetes did not become insulin-resistant. Studies have shown that hyperglycemia can modulate insulin signaling by impairing insulin receptor activity and Pi3K/AKT signaling (13). Nonetheless, Ordóãez et al. (13) proposed that hyperglycemia has different effects depending on the exposure time, and that a prolonged exposure time leads to insulin resistance, whereas a short exposure time does not. Therefore, we believe that the duration of hyperglycemia in the current study was not sufficient to promote insulin resistance.

Nevertheless, STZ-induced diabetic mice presented classic disease symptoms, such as polyphagia, polydipsia, and weight loss (Figure 1D-H). Soon after SZT induction, the animals began to lose weight rapidly, showing a catabolic state typical of severe hyperglycemia. The catabolic pathway breaks down molecules to generate energy. As glucose uptake by adipocytes and muscle cells does not occur properly, the body is pushed to activate the catabolic program and shift from glucose to lipid metabolism, leading to the release of lipids and free fatty acids (FFAs) to provide energy (14). In addition, increased plasma concentrations of glycerol and FFAs provide a substrate for hepatic gluconeogenesis, which increases blood glucose alarmingly, leading to osmotic diuresis and consequently polydipsia (15). Diabetic mice treated with SZT are hyperphagic (16). The hyperphagia in diabetic animals is accompanied by hyperglycemia and elevated plasma levels of ghrelin (16). Ghrelin is mostly produced in the stomach in response to fasting and circulates in the blood. Ghrelin affects the control of food intake by modulating neuropeptide Y (NPY) in the hypothalamus. This phenomenon explains the increased hunger in the weight loss state caused by hyperglycemia in this model (17).

Many metabolic changes during hyperglycemia also affect the skin, and 30-70% of individuals with diabetes develop skin diseases (18). The molecular mechanisms for the development of these diseases are not yet fully elucidated (19). Diminished epidermal lipid production and lamellar body synthesis, and decreased hydration of the stratum corneum have been associated with chronic hyperglycemia, leading to deterioration of the cutaneous barrier function, and making the skin susceptible to dryness and infections (7,20). Furthermore, increased oxidative stress, low-grade inflammation, and increased transcription factors are important features associated with the development of skin lesions due to diabetes (5). Thus, in our animal model, we observed some modulations in the transcripts of important pro- and anti-inflammatory mediators and in growth factors that could result in alterations in the uninjured skin of diabetic mice (Figure 2).

TNF-α is a pro-inflammatory cytokine that is crucial for regulating the inflammatory process during cutaneous wound healing (21,22). Continuously high levels of TNF-α can prolong the inflammatory phase and inhibit healing (22). Also, high levels of TNF-α in human dermal fibroblast culture result in premature senescence, leading to a reduced proliferative potential (23). Therefore, elevated concentrations of TNF-α in a microenvironment, as observed here, could lead to tissue alterations (Figure 2A). Moreover, IL-6 is increased in chronic wounds of humans and animals (22). IL-6 is a pleiotropic cytokine with diverse biological actions (24). It plays a crucial role in the pathogenesis of local and systemic inflammation in the skin, in autoimmune diseases, and in tumor development (24). Surprisingly, we observed a decrease in IL-6 mRNA in our study (Figure 2C). It has been reported that keratinocytes are the main skin cells responsible for the production and release of IL-6 (25). Perhaps the decrease in the number of keratinocytes and the change in their morphology described in this study explain the decrease in IL-6 mRNA in the skin of diabetic mice (Figure 3A and B).

F4/80 is a glycoprotein known as one of the most specific markers for murine macrophages, including Langerhans cells in the skin (26). Both resident and recruited macrophages are primarily responsible for establishing and maintaining skin homeostasis (27). They play important roles in wound repair, follicular regeneration, regulation of inflammation, autoimmunity, and skin cancer (27). In diabetes, the actions of macrophages are modified, exhibiting hyperresponsiveness to inflammatory stimuli, enhanced secretion of pro-inflammatory cytokines, and reduced ability to phagocytose pathogens and dead cells (28). Our data suggested that macrophages were increased in the uninjured skin of diabetic mice, but that they may not function properly, increasing pro-inflammatory cytokines (Figure 2E). Other studies have shown that there is low-grade inflammation in the uninjured skin of individuals with diabetes (29), corroborating our findings. Interestingly, we observed an increase in anti-inflammatory cytokines, such as *Il-10* and *Tgf-β1* (Figure 2D and G). The increase in anti-inflammatory mediators combined with decreased levels of *Il-6* could indicate the presence of compensatory counter-regulation of pro-inflammatory stimuli in this model (30).

Insulin-like growth factor 1 (*IGF-1*) transcripts are highly expressed in several tissues of the body and its expression in the liver is regarded as the main source of IGF-1 in the blood (https://www.genecards.org/cgi-bin/carddisp.pl?gene=IGF1&keywords=igf-1). However, the protein expression of IGF-1 is highest in the skin (https://www.genecards.org/cgi-bin/carddisp.pl?gene=IGF1&keywords=igf-1). In this study, we showed that the uninjured skin of diabetic mice expressed even higher levels of *Igf-1* than that of non-diabetic mice (Figure 2I). The high levels of *Igf-1* in the skin of mice with diabetes overlapped with those of well-known skin abnormalities caused by elevated plasma concentrations of IGF-1, such as acanthosis nigricans, dermatosis of body folds, and oily skin with keratosis (31). Moreover, skin IGF-1 could also contribute to the increased levels of plasma IGF-1 present in type 2 diabetes mellitus.

Chronic hyperglycemia induces harmful biological processes that could promote extracellular matrix (ECM) modifications, such as fibrosis, inflammation, and pathological angiogenesis (32). Here, in the H/E analyses, we observed a thinner epidermis with a lower cellular density in diabetic mice, with smaller and fewer keratinocytes than in control mice (Figure 3A-D). These data corroborate studies by Sakai et al. (20), which showed a thinner epidermis in STZ animals with diminished proliferation of keratinocytes. Moreover, we noticed a diminished number of dermal cells in the skin of diabetic mice (Figure 3E). The most predominant cells in the dermis are fibroblasts. Studies have shown that hyperglycemia impairs the proliferation of dermal fibroblasts in culture, which could explain our results. Fibroblasts are critical for effective wound healing, being responsible for secreting collagen, which is the main structural protein of the skin. Changes in collagen significantly contribute to connective tissue aging and imbalance in skin homeostasis (33). It has been well documented that nascent collagen undergoes various post-translational modifications, both intracellularly and extracellularly, called maturation. Mature collagens are sufficiently mechanically stable to maintain cell propagation (34). In our study, although the skin of diabetic mice showed an increase in the quantity and uniformity of total collagen fibers, the collagen fibers were less organized, in addition to being more immature (Figure 3H-K). These findings may explain some of the cutaneous wound healing problems, such as the proliferation of fibroblasts, that occur in diabetic individuals. TGF-β1 can induce collagen deposition, controlling both the deposition and turnover of ECM elements such as fibrillary collagen and fibronectin and inhibiting the expression of matrix-degrading proteolytic enzymes such as serine proteinases and MMPs (35,36). Collagen types I and III are synthesized during wound healing, although type III collagen production decreases much earlier during epithelialization (36). Studies support a role for TGF-β1 as a mediator of both type I and type III collagens (37). So, the increase in *Tgf-β1* and the trend for a reduction in *Mmp-9* observed here could explain in part the increase in type III immature collagen observed in the skin of diabetic mice.

The alterations caused by hyperglycemia also affect peripheral neurons and the expression of neurotrophic factors (38). Brain-derived neurotrophic factor (BDNF) plays an important role in neuronal survival and growth and may also act as a trophic factor in the epithelium, being intimately involved in the control of epidermal homeostasis (39). Transgenic mice that overexpress BDNF had a significantly thicker epidermal layer and an increased number of Ki-67 positive epidermal keratinocytes (*in vivo*), while BDNF knockout mice had fewer of these cells (39). We found a decrease in the transcripts and protein expression of BDNF and no changes in its receptor in the skin of diabetic mice (Figure 4A-C). This phenomenon could also explain the alterations observed in the morphology of the epidermis in the diabetic mice. Following the decrease in BDNF mRNA, there was a significant decrease in the number of peripheral neurons in diabetic mice via Nissl staining, as expected (Figure 4D and E). These data showed once again important changes caused by hyperglycemia that disrupt skin homeostasis even before the appearance of wounds and hinder healing.

In conclusion, this work showed that chronic hyperglycemia *per se* causes disruption in skin homeostasis in uninjured tissue, leading to morphological, cellular, and molecular alterations that lead to skin diseases and impaired wound healing. The alterations observed in the expression of *Tnf-α*, *F4/80*, *Il-6*, *Tgf-β1*, *Igf-1*, and BDNF could predispose to the disruption of uninjured skin homeostasis, suggesting that these alterations are the primary cause of impaired wound healing in diabetes. These findings will help professionals better understand skin lesions in diabetic patients and should be used to encourage patients to better adhere to their treatment even before the development of diabetic complications.
